# Applicability of the hour of life approach in hyperbilirubinemia among Filipino term infants

**DOI:** 10.3389/fped.2022.990919

**Published:** 2022-10-13

**Authors:** Maria Esterlita T. Villanueva-Uy, Herbert G. Uy, Maria Lourdes E. Amarillo

**Affiliations:** ^1^Institute of Child Health and Human Development, National Institutes of Health, University of the Philippines Manila, Manila, Philippines; ^2^Philippine General Hospital, University of the Philippines Manila, Manila, Philippines; ^3^Institute of Clinical Epidemiology, National Institutes of Health, University of the Philippines Manila, Manila, Philippines

**Keywords:** hyperbilirubinemia, neonatal, nomogram, Filipino, transcutaneous bilirubin, risk factors

## Abstract

**Background:**

Hyperbilirubinemia remains a common morbidity among infants. Additional research on bilirubin kinetics and associated risk factors will contribute to providing a more targeted management approach for the Filipino infant.

**Objective:**

To develop a Filipino bilirubin nomogram by studying bilirubin patterns during the first 5 days of life.

**Methodology:**

This prospective study recruited 1,412 stable, full-term infants (≥37 weeks age of gestation) born at the Philippine General Hospital (PGH). Using the Dräger-Minolta JM-103 jaundice meter, transcutaneous bilirubin (TcB) levels were determined at the 3rd, 6th, 12th, 24th, 36th, 48th, 72nd, 96th, and 120th hour of life (HOL). A bilirubin nomogram was created using the averages of 3 TcB forehead and sternal measurements at each time epoch. Simultaneous measurement of TcB and total serum bilirubin (TsB) on a subset of 106 infants was done to determine correlation.

**Results:**

Correlation coefficients were high between TsB and forehead TcB (r^2^ = 0.88), and between TsB and sternal TcB (r^2^ = 0.91). The Filipino bilirubin nomogram reflected a steep rise until the 48th hour, followed by plateauing of values. Inadequate nursing and bilirubin levels at 12th and 48th HOL were risk factors for developing significant hyperbilirubinemia at 72nd HOL.

**Conclusion:**

TcB is a reliable, non-invasive bilirubin screening tool. Among healthy, full-term, Filipino infants, their nomogram features a sudden increase in bilirubin values during the first 48 h, followed by a plateau. To aid in identification of infants at risk for significant hyperbilirubinemia, healthcare providers can assess breastfeeding adequacy and perform bilirubin screening at the 24th−48th HOL. Registration No. (RGAO-2016-0686).

## Introduction and background

Hyperbilirubinemia, manifested by yellowish skin color, is the most common morbidity in the newborn period (1). The worldwide resurgence of kernicterus—the consequence of severe hyperbilirubinemia—elicited concern for this morbidity. Mortality rate can reach as high as 10% with 70% of the survivors having sequelae of kernicterus (2). Ironically, kernicterus has been described as the most easily preventable form of brain injury in the neonatal period. This has been attributed to delay in recognition and delivery of optimal treatment, especially in low resource settings (3).

The incidence of neonatal hyperbilirubinemia in high income countries has been reported from large country databases. However, the incidence of neonatal jaundice in low to middle income countries is variable since classifications may have been established in the local level, absence of a unified protocol and data obtained mostly from tertiary hospitals (4). Furthermore, early discharge is practiced with clinical follow up not assured, further increasing the risk in the development of severe hyperbilirubinemia (5). Societal awareness of the incidence and complication of neonatal hyperbilirubinemia especially in LMIC countries needs to be strengthen by both pre-discharge and community surveillance (6). Additional risk factors for the development of significant hyperbilirubinemia in low to middle income countries include maternal factors (primiparous, delivery outside the hospital) and neonatal factors (lower gestational age and birth weight, UDP glucuronosyltransferase 1 family, polypeptide A cluster (UGT1A) polymorphisms and sepsis) (7).

In 2004, the American Academy of Pediatrics (AAP) published the hyperbilirubinemia guidelines to better monitor, manage, and follow-up all newborn infants (8). AAP adapted the Hour of Life Approach strategy by Bhutani (9), which utilized a graph identifying risk zones based on the serum bilirubin levels at specific time epochs. This made risk assessment a dynamic process rather than dependent on a single bilirubin level to identify at-risk infants. Unfortunately, only 4.1% of Bhutani's infant population were of Asian descent—a race with a higher incidence of hyperbilirubinemia. Several studies have shown that Asian neonates reach earlier bilirubin peaking as well as higher total bilirubin values (10–12). As such, it is important that each population develop its own nomogram (11). Bilirubin nomograms tailored to certain populations have already been developed: Italian, Greek, American, Hispanic, Brazilian, Indian, Japanese, Thai, and Chinese (13–20). These nomograms showed that some races have higher 95th percentile bilirubin values especially in the first 3 days of life.

Filipinos are believed to have a higher risk of developing significant hyperbilirubinemia (SH). Risk factors, as listed in the AAP guidelines (8), found among Filipino infants include belonging to the East Asian race, high rates of early breastfeeding initiation, and high incidence of Glucose-6-Phosphate Dehydrogenase (G6PD) deficiency (21). While Filipino infants have been included in hyperbilirubinemia studies, they rarely comprise the majority and often grouped under Asian (22) or Pacific Islander descent (23). Studying the bilirubin kinetics among Filipino infants will help in the early recognition and management of significant hyperbilirubinemia.

## Objective

This study aimed to develop a Filipino bilirubin nomogram by studying the bilirubin pattern in the first 5 days of a Filipino infant's life.

## Significance of the study

There is a dearth of information regarding bilirubin kinetics in the Philippines. In the attempt to develop a Filipino-based bilirubin nomogram, this study will provide new information regarding the reliability of the transcutaneous bilirubin measurement among Filipino infants. Additionally, it also paves the way for identification of specific risk factors for developing significant hyperbilirubinemia centered on the Filipino population.

The eventual development of an hour of life nomogram specific for the Filipino infants will help pediatricians monitor, manage, and follow-up infants at risk of significant hyperbilirubinemia. Moreover, clinical guidelines on management of hyperbilirubinemia may be submitted to the national societies for guidance and implementation.

## Methodology

### Study design

This is a prospective cohort study which recruited stable full term Filipino infants after birth and monitored bilirubin levels at specific time points up to 120 h of life of stable, with the eventual output of a Filipino bilirubin nomogram.

### Study setting

Recruitment of participants was conducted at the Philippine General Hospital (PGH). PGH is a tertiary referral center which is the training hospital of the University of the Philippines. The annual live births in the hospital is around 4,000–6,000 per year. It has a tertiary level NICU with an accredited Fellowship training program in Newborn Medicine. Daily NICU census ranges from 40–60 sick neonates. There are two maternity wards (total of 76 beds) where the mothers and their stable infants are admitted.

### Study population and sampling design

The study used convenient sampling of all eligible infants. The research assistants approached parents of newly born infants who met the following requirements:

Inclusion criteria:

1. Stable, full term (≥37 weeks) newborn infants.
2. <3 h old.
3. Assigned for direct rooming in with mothers in maternity wards.

Exclusion criteria:

1. Small for gestational age infants.
2. Infants who were not given oral feeding.
3. Infants with gastrointestinal anomalies.
4. Infants with lethal congenital anomalies.

### Sample size computation

A sample size of at least 1,641 observations was computed with a power of 80% and a 5% level of significance with ABO incompatibility as an independent variable (38% Blood type O and 62% non-Blood Type O) and allowing for a change from a baseline value of 10–20%. This change was correspondent to an odds ratio of 2.25.

The study also correlated transcutaneous bilirubin and serum bilirubin measurements. Based on Ho et al.'s (24) study, with desired r = 0.1, power of 0.80, and α of 0.05, at least 83 newborn infants were needed.

### Study plan

#### Definition of terms

Significant hyperbilirubinemia (SH) was defined as having total bilirubin levels ≥95th percentile and would require interventions such as phototherapy (9).

Severe hyperbilirubinemia was defined as having total bilirubin levels reaching ≥99th percentile and would require interventions such as double volume exchange transfusion, aside from intensive phototherapy (8).

#### Conduct of the study

A trained research assistant approached the mothers whose infants were roomed-in with them in the maternity wards. All infants admitted in the maternity wards were stable infants on exclusive breastfeeding. Once the infant was assessed to meet the inclusion criteria, a written informed consent was obtained from the mother or a legally appointed representative, in case the mother could not provide an informed consent. After an informed consent is obtained, pertinent maternal and infant data were extracted from the medical records. Feeding, urine and stool frequencies were also extracted from the daily monitoring forms in the medical chart. The mothers were approached in case of any clarifications or missing data in the chart.

#### Transcutaneous (TcB) bilirubin measurement

The research assistants underwent orientation and training on how to use the transcutaneous bilirubinometer, the Dräger-Minolta JM-103 jaundice meter (25). The device determined the yellowness of the infant's subcutaneous tissues through differential measurement of optical densities using blue and green wavelengths. Using two optical paths allowed for more precise measurement of the jaundiced subcutaneous tissues. This was due to the decreased influence of melanin movement and skin maturity.

The device was placed perpendicularly on the forehead and the sternum. Selection of these sites were based on manufacturer's recommendation, based on the principle of sufficient circulation to these areas. The device was pressed gently against the infant's skin until a click was heard. A reading would be shown on the screen. Three determinations were made for both the forehead and the sternum for a total of six determinations per pre-specified time period. Transcutaneous bilirubin (TcB) measurements were done at the 3rd, 6th, 12th, 24th, 36th, 48th, 72nd, 96th, and 120th hour of life (HOL). The TcBs obtained at the different time periods were used to develop the bilirubin nomogram. The device was wiped with 70% alcohol between patients.

#### Total serum bilirubin (TsB) measurement

To correlate total serum bilirubin with transcutaneous bilirubin values, 106 infants had simultaneous determinations of the TcB bilirubin (average of three determinations of both the forehead and the sternum) and total serum bilirubin (TsB). For each patient, 0.5 ml of venous blood was extracted by trained healthcare professionals. These specimens were placed in a plain microtainer covered with a black carbon paper and sent immediately to the laboratory for analysis. The VITROS XT7600 and 5600 models were used to determine total bilirubin levels in the subset of neonates.

### Statistical analysis

The data were encoded in MS Excel. All statistical tests were performed using the IBM Statistical Package for the Social Sciences (SPSS) software. Descriptive statistics were used to summarize demographic profiles. Frequency and proportion were used for categorical variables. Mean and standard deviation were used for normally distributed continuous variables.

Pearson correlation coefficient was computed to correlate TsB and TcB readings. For the development of the bilirubin nomogram, TcB quartiles were determined at the specified time periods. The 95th and the 99th percentile were also determined for significant and severe hyperbilirubinemia, respectively. The 40th centile which was equivalent to the cut off for the low-risk zone in the Bhutani bilirubin nomogram, was also determined.

For the maternal and neonatal risk factors in the development of significant hyperbilirubinemia at the 72nd HOL, independent t-test and Chi square test were used for continuous and categorical variables.

### Ethical consideration

Before the start of the study, Ethics Review Board (ERB) approval was secured (UPMREB Code: 2009-018-01). At the maternity wards, written informed consents were provided and explained to the infants' respective parents by trained research assistants. Participation was completely voluntary; participants were given the right to withdraw consent at any time. All data were anonymized. Data forms were stored in locked cabinets. Data were encoded in password protected computers with access limited to the investigators.

## Results

### Study population demographics

One thousand four hundred and twelve stable, full-term infants, including 36 pairs of twins, were recruited from 2010 to 2014. The infants had the following mean anthropometric measurements: birthweight of 2,872 ± 0.45 g, length of 48.0 ± 2.71 cm, and head circumference of 33 ± 1.7 cm. Most infants were delivered vaginally (61.9%), in cephalic presentation (92.9%), with APGAR scores of 8 becoming 9 at the 1st and 5th minutes of life, respectively.

Mean maternal age was 30.6 ± 7.46 years old; 65.8% was of single civil status. 99.2% received prenatal care with an average prenatal visit of 1.7 ± 0.464 times. Maternal morbidities were as follow: premature rupture of membranes (PROM) (6.2%), bleeding placenta previa (0.9%), diabetes (18.2%), and history of infection (26.8%). Twenty-one mothers (2.9%) were smokers; 15 (2.1%) were alcohol drinkers and 6 (0.6%) mothers admitted to illicit drug use.

All infants were exclusively breastfed at the hospital, with an average of 9.3 ± 2 breastfeeding episodes for the first 24 h. During the first day of life, the average number of urination and stooling per day were 1.5 ±0.91 and 1.8 ± 0.95, respectively.

### Correlation of total serum bilirubin and transcutaneous bilirubin

**Figure 1:**
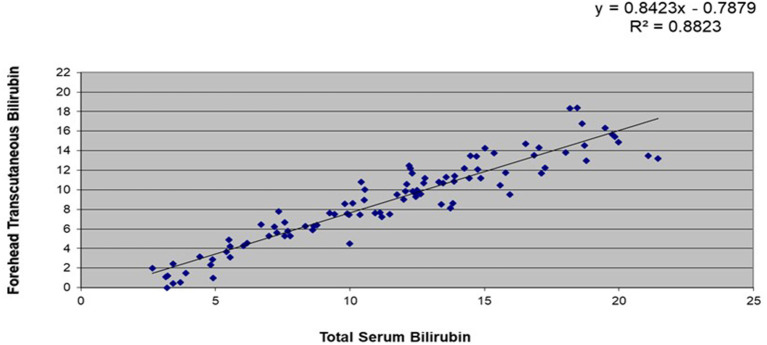
Correlation between total serum bilirubin (TsB) and forehead transcutaneous bilirubin (TcB) levels among stable, full-term Filipino infants (*n* = 106).

**Figure 2:**
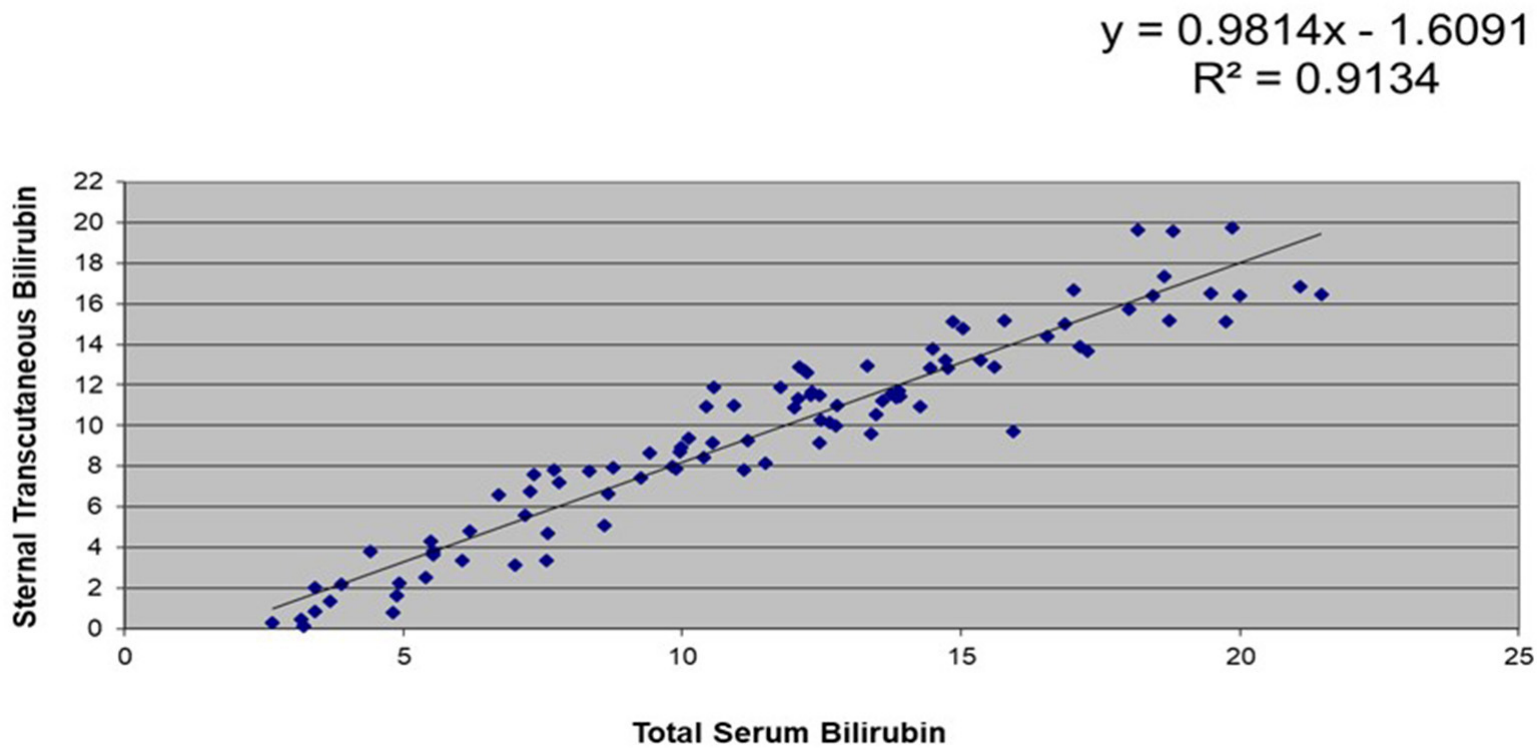
Correlation between total serum bilirubin (TsB) and sternal transcutaneous bilirubin (TcB) levels among stable, full-term Filipino infants (*n* = 106).

A subset of 106 stable, full-term infants had data for simultaneous TsB and TcB levels. Correlation coefficients between TsB and forehead TcB was at R^2^ = 0.88, while those between TsB and sternal TcB was R^2^ = 0.91 (refer Figures 1, 2).

### Mean bilirubin values at different time points

Table 1 showed the mean bilirubin levels at different hours of life. Among infants who subsequently developed SH at the 72nd HOL, the mean bilirubin values were significantly higher than those who did not. There was almost a doubling (1.6–1.8-fold increase) in mean TcB values from 3rd to the 6th hours, 6th to the 12 hour and 12th to the 24th hour. There was a fivefold increase for the first 24 h (3rd HOL to 24th HOL) which slowed down from 24th to 48th HOL (1.3–1.56-fold increase) (see Table 1).

**Table 1:** Comparison of mean bilirubin levels at different time points and subsequent high risk zone status at the 72nd HOL.

**Time points**	<**95**th **percentile at 72**nd **HOL**		≥**95**th **percentile at 72**nd **HOL**		***p value***
	***n***	**Mean TcB**	***n***	**Mean TcB**	
3^rd^ HOL	1370	1.4 ± 0.87	42	2.1 ± 1.09	0.00
6th HOL	1370	2.5 ± 1.18	42	3.6 ± 1.24	0.00
12th HOL	1370	4.2 ± 1.35	42	5.6 ± 1.62	0.00
24th HOL	1281	7.4 ± 2.64	42	9.3 ± 2.87	0.00
48th HOL	1077	9.8 ± 2.87	42	14.7 ± 2.27	0.00

### Determination of different bilirubin percentile groupings at different time points

One thousand four hundred and twelve infants had TcB values at the 3rd, 6th, and 12th HOL. Infants were discharged around the 24th HOL which would explain attrition in the number of TcB determinations in the subsequent time points. The mothers were encouraged to return for subsequent determinations but not all returned despite calling or sending text messages. There was a total of 6 TcB determinations (3 on the forehead and 3 on the sternum) in each infant. Subsequently, there were 8,472 TcB determinations (*n* = 1,412 infants) each for the 3rd, 6th, and 12th time period, 7,938 TcB determinations (*n* = 1,323 infants) for the 24th HOL, 6,714 (*n* = 1,119 infants) for the 48th HOL, 5,136 (*n* = 856 infants) for the 72nd HOL, 3,630 (*n* = 605 infants) at the 96th HOL and 2,388 (*n* = 308 infants) at the 120th HOL. Bilirubin values were enumerated into 25th, 40th, 50th, 75th, 95th and 99th percentiles.

#### Determination of the cut-off for the low-risk zone

##### <*25th percentile*

The chances of TcB levels <25th percentile at different time points shifting to levels >95th percentile (High risk zone or HRZ) on subsequent time points were from 0 to 4.6% (see Table 2). Upon further analysis, the risks of bilirubin values ≤ 25th percentile shifting to the high intermediate risk zone (HIRZ = 75th to the 95th percentile) in subsequent time periods were low at 3.1–8.1%.

**Table 2:** Comparison of different bilirubin percentiles (25th, 40th and 50th) and frequencies of developing significant hyperbilirubinemia (≥ 95th percentile) at different time points.

**HOL**	**Percentiles**	**Frequency (%) of significant hyperbilirubinemia at different time periods**				
		**12th HOL**	**24th HOL**	**48th HOL**	**72nd HOL**	**96th HOL**
3^rd^	25th	0.6	4.6	0.6	0.9	0.8
	40th	2.0	3.9	0.7	1..3	1.1
	50th	2.0	4.0	1.1	1.6	1.4
6th	25th	1.3	3.7	0.6	0.8	0.
	40th	2.0	2.7	0.7	1.1	1.2
	50th	2.1	2.8	1.1	1.3	1.1
12th	25th		4.6	0.6	1.1	1.7
	40th		4.5	1.0	1.6	1.6
	50th		3.8	1.1	1.5	1.5
24th	25th			0.8	1.2	1.8
	40th			0.6	1.3	1.7
	50th			0.6	1.4	1.5
48th	25th				0	0
	40th				0	0.4
	50th				0.2	0.9

##### <*40th percentile*

TcB levels <40th percentile at different time points have risks of significant hyperbilirubinemia on subsequent time points ranged from 0 to 4.5% (see Table 2). The risks of shifting to TcB levels between 75 and 95th percentile (HIRZ) were 6.6–9.1%.

##### <*50th percentile*

TcB levels <50th percentile have risks of significant hyperbilirubinemia at subsequent time periods from 0.2 to 3.8% (see Table 2). The risk of shifting to TcB levels between 75 and 95th percentile was 0.1–11.1%.

### Percentile groups and risk of upward shift

#### 25th−50th percentile

Taking bilirubin levels between the 25th−50th percentiles revealed a risk of developing SH at subsequent time periods to be from 2.1 to 4.9% only. However, the risks of shifting to HIRZ were high at 19.9, 18.6, 15.7–16.8, and 12.1–15.3% at 12th, 24th, 48th and 72nd HOL. The risk of shifting to HIRZ at the 96th HOL for bilirubin values from 25 to 50th percentile were 8.5 to 10.7% (see Table 3).

**Table 3:** 25–50th percentile bilirubin levels and subsequent shift to higher percentile groups (75– <95th and ≥ 95th) at different time points.

**25-50th percentile**	**Frequency (%) of shifting to higher risk zones at subsequent time points**									
	**12**th **HOL**		**24**th **HOL**		**48**th **HOL**		**72**nd **HOL**		**96**th **HOL**	
	**75- <95th**	**≥95th**	**75- <95th**	**≥95th**	**75- <95th**	**≥95th**	**75- <95th**	**≥95th**	**75- <95th**	**≥95th**
3^rd^ HOL	19.9	4.9	18.6	4.7	15.7	3.9	12.1	3	8.5	2.1
6th HOL	19.9	4.9	18.6	4.7	15.7	3.9	12.1	3	8.5	2.1
12th HOL			18.6	4.7	15.7	3.9	12.1	3	8.5	2.1
24th HOL					16.8	4.2	12.9	3.2	9.1	2.3
48th HOL							15.3	3.8	10.7	2.7

#### 50th−75th percentiles

For TcB levels between the 50th and the 75th percentiles, the risk of developing SH was <5% on subsequent time periods. While shifting to the HIRZ were 19.9, 18.6, 15.7–16.8, and 8.5–10.7% at 24th, 48th, 72nd, and 96th HOL.

#### 75th−95th percentile

Values at the HIRZ remained stable overtime with only <5% shifting to High-Risk Zone.

### Plotting the selected bilirubin percentiles

**Figure 3:**
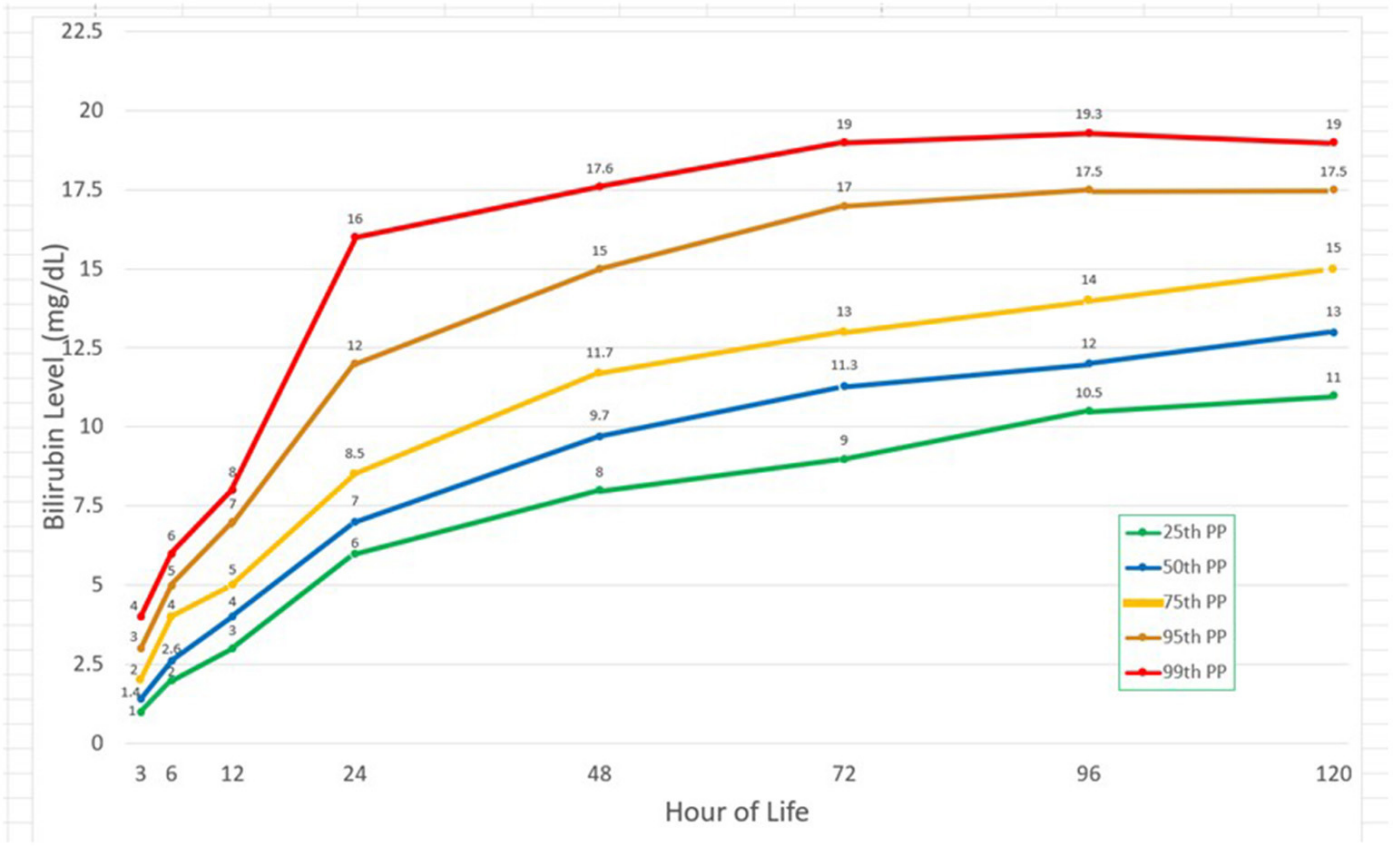
Bilirubin values at different time points and corresponding 25th, 50th, 75th, 95th, and 99th percentiles.

The graph was plotted using the first three quartiles and then the 95th and the 99th percentile. In Figure 3, there was a steep rise in the bilirubin from the 3rd to the 24th hour of life with doubling of the bilirubin levels from the 3rd hour of life to 6th hour of life as well as from the 12th to the 24th HOL. After the 24th HOL, there was around 2–3 mg/dl increase in the bilirubin levels at the 48th HOL. From the 48th HOL to the 72nd HOL, the increase was from 1 to 2 mg/dl only. From 72nd to 120th HOL, the 95th and 99th percentile TcB levels have plateaued. For the 25th, 50th and 75th percentile bilirubin levels, there remained a 1 mg/dl/day increase from the 72nd to the 120th HOL but the values did not reach 17 mg/dl (significant hyperbilirubinemia).

### Risk factors for developing significant hyperbilirubinemia at 72nd HOL

AAP listed risk factors for developing significant hyperbilirubinemia (SH). One of which was of East Asian descent and in this study, all participants were Filipinos. Significant AAP risk factors were presence of bruising (*p* = 0.001) and cephalhematoma (*p* = 0.001) while male sex (*p* = 0.442), younger gestational age (*p* = 0.227), and history of jaundice in an older sibling (*p* = 0.772) were not significant (see Table 4).

**Table 4:** Association of AAP guidelines' risk factors and significant hyperbilirubinemia at 72nd HOL.

**Risk factors**	**No Significant hyperbilirubinemia**	**Significant hyperbilirubinemia**	***p* values**
	**at 72nd HOL**	**at the 72nd HOL**	
1. East Asian race	All Filipino descent	
2. Exclusive breastfeeding	All were exclusively breastfed	
3. G6PD deficiency (patient or relative	Results not available	
4. Sex			0.581
Male	679	19	
Female	691	23	
5. Jaundice in a previous sibling			0.087
Yes	2	1	
No	1,370	41	
6. Cephalhematoma			0.001^*^
Yes	4	3	
No	1,366	39	
7. Bruising			0.012^*^
Yes	14	3	
None	1,356	39	
8. Mother's blood type			0.765
Blood Type O	866	26	
Other Blood types	489	16	

* For cells with <5 numerical values, Fischer's Exact test was performed. For the rest, Pearson's Chi-square was done.

Additional significant risk factors for developing SH at the 72nd HOL were <3 prenatal care visits (*p* = 0.026), unspecified maternal infection aside from UTI (*p* = 0.008), and TcB readings >95th percentile at the 12th and the 48th HOL. Non-significant risk factors were maternal blood type O^+^ (*p* = 0.304), jaundice at the first 24 hours (*p* = 0.772), fetal presentation (*p* = 0.093), route of delivery (*p* = 0.784), PROM (*p* = 0.078), maternal diabetes (*p* = 0.643), maternal UTI (*p* = 0.187) and histories of tobacco smoking (*p* = 1.0), alcohol (*p* = 1.0) and substance abuse (*p* = 0.619) (see Table 5).

**Table 5:** Association of maternal characteristics and significant hyperbilirubinemia at the 72nd HOL.

**Characteristics**	**No Significant hyperbilirubinemia at 72nd HOL**	**Significant hyperbilirubinemia at the 72nd**	***p* values**
Marital status			0.50
Single	648	15	
Married	450	16	
Prenatal care			0.26
Yes	1,381	41	
No	9	1	
No of prenatal visits			0.026^*^
<3 visits	389	21	
3 and above	978	21	
History of chorioamnionitis			1.0
Yes	1,369	42	
No	1	0	
Presentation			0.093
Cephalic	1,264	40	
Traverse	88	2	
Breech	88	2	
Route of delivery			0.784
Vaginal	783	23	
Forceps	73	4	
Vacuum	40	1	
Cesarean section	514	14	
PROM (>18 hours)			0.078
None	1,293	38	
Yes	77	3	
Antibiotic use			0.51
Yes	658	18	
No	712	24	
History of diabetes			0.643
No	1,137	36	
Yes	233	6	
Amniotic fluid volume			0.001^*^
Normal	1,357	39	
Oligohydramnios	13	3	
History of infection			0.001^*^
Yes	345	20	
No	1,025	22	
UTI			0.187
Yes	336	16	
No	499	17	
Substance abuse			0.619
Yes	8	0	
No	1,362	42	
Tobacco usage			1.0
Yes	21	0	
No	775	28	
Alcohol use			1.0
Yes	18	0	
No	780	28	

* For cells with <5 numerical values, Fischer's Exact test was performed. For the rest, Pearson's Chi-square was done.

All the infants were breastfed. For the feeding patterns, decreased feeding frequency at all time points except at first 24 hours, decreased urine frequency at 12th to 48th HOL, and decreased stool frequency from 12th to 24th HOL were all significant risk factors (see Table 6).

**Table 6:** Association of feeding pattern and significant hyperbilirubinemia at 72nd HOL.

	**No significant hyperbilirubinemia at 72**nd **HOL**		**Significant hyperbilirubinemia at 72**nd **HOL**		***p value***
	***N***	**Mean ±SD**	***N***	**Mean ±SD**	
3^rd^ HOL
Weight	1368	2865 ± 446.8	42	2740 ± 432.5	0.074
Feeding frequency	1370	0.6 ± 0.6	42	0.7 ± 0.44	0.042^*^
Urine frequency	1370	0.1 ± 0.29	42	0.02 ± 0.15	0.103
Stool frequency	1370	0.1 ± 0.11	42	0.2 ± 0.15	0.627
6th HOL
Weight	1366	2845 ± 445.8	42	2740 ± 432.5	0.073
Feeding frequency	1370	1.3 ± 1.11	42	1.8 ± 0.76	0.001^*^
Urine frequency	1370	0.90 ± 0.85	42	0.95 ± 0.53	0.730
Stool frequency	1370	0.70 ± 0.62	42	0.8 ± 0.45	0.284
12th HOL
Weight	1368	2865 ± 446.8	42	2739 ± 432.5	0.074
Feeding frequency	1370	3.12 ± 3.21	42	4.28 ± 2.29	0.020^*^
Urine frequency	1370	1.21 ± 1.01	42	1.90 ± 0.69	0.001^*^
Stool frequency	1370	0.8 ± 0.75	42	1.4 ± 0.58	0.001^*^
24th HOL
Weight	1370	2866 ± 446.3	42	2740 ± 432.7	0.071
Feeding frequency	1247	9.3 ± 1.99	42	9.3 ± 2.0	0.885
Urine frequency	1365	1.6 ± 0.93	42	2.17 ± 1.05	0.001^*^
Stool frequency	1365	1.8 ± 0.94	42	2.1 ± 0.92	0.029^*^
48th HOL
Weight	1075	2842 ± 454.5	42	2739 ± 432.7	0.151
Feeding frequency	1075	9.8 ± 1.80	42	10.6 ± 1.51	0.004^*^
Urine frequency	1075	2.6 ± 0.98	42	3.2 ± 0.75	0.001^*^
Stool frequency	1075	2.2 ± 0.82	42	2.3 ± 0.74	0.788

### Finalizing the Filipino bilirubin nomogram

**Figure 4:**
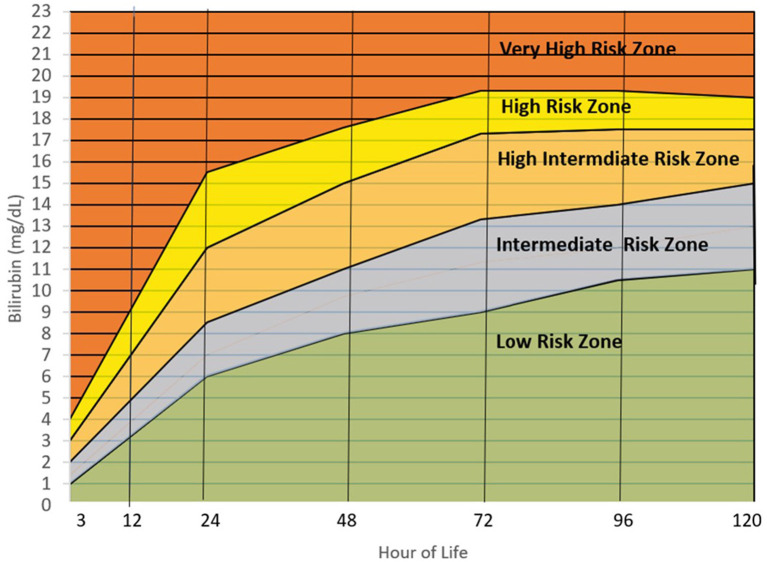
Filipino bilirubin nomogram showing the different risk zones. Low Risk Zone or LRZ (0– <25th percentile)-Infants with bilirubin levels falling in this zone have a <5% risk of developing significant hyperbilirubinemia (SH) (>95th percentile or High-risk zone). The infants may be discharge and followed up after 48–72 hours, especially if they were discharged <72 hours of life. Intermediate Risk Zone or IRZ (25th– <75th percentile)-Infants with bilirubin levels in the IRZ have a <5% risk of subsequent SH but have a 15–20% risk of shifting to the High-Risk Intermediate Zone (HIRZ), especially if the bilirubin determination was done in the first 48 hours. For infants with risk factors, bilirubin may be repeated after 24 hours. High Intermediate Risk Zone or HIRZ (>75th– <95th percentile)-Infants with bilirubin levels in the HIRZ, should be further observed and have a repeat of bilirubin determination after 24 hours. In the presence of risk factors such as hemolytic disease, young gestational age, weight loss (>10%), sepsis and others, phototherapy may be started. High Risk Zone or HRZ (95th– <99th percentile)-Infants with bilirubin levels at the HRZ have significant hyperbilirubinemia and require initiation of phototherapy. Very High Risk Zone or VHRZ (≥99th percentile)-Infants with bilirubin levels at the VHRZ have severe hyperbilirubinemia and require immediate intensive phototherapy while preparing for exchange transfusion. Risk factors identified by the AAP and significant in this study are: Gestational age < 38 weeks, inadequate nursing (significant weight loss), male sex, ABO/RH incompatibility, G6PD deficiency, East Asian race, cephalhematoma/bruising, previous sibling requiring phototherapy during the neonatal period. Other risk factors identified in this study are: Inadequate prenatal visits, maternal infection, decreased amniotic fluid, and decreased feeding, urine, and stooling frequency in the first 72 hours.

Figure 4 is the Filipino bilirubin nomogram with the different risk zone categories. Low Risk Zone (LRZ) which contains bilirubin values <25th percentile, has a <2% risk of developing significant hyperbilirubinemia (>95th percentile or High Risk Zone) at all time points except at the 24th HOL when the risk is at 4.6%. For the Intermediate Risk Zone (IRZ), the 2nd and 3rd quartiles (25th–75th percentiles) have been combined due to having the same risk of developing significant hyperbilirubinemia. The IRZ has a <5% risk of developing SH but an almost 20% risk of shifting to the next zone, the High Intermediate Risk Zone (HIRZ), especially during the first 24 hours of life. The HIRZ has a <5% risk of shifting to the High Risk Zone (HRZ). Still, the HIRZ may be used as a cut-off for starting phototherapy among infants with risk factors. Bilirubin levels in the HRZ (>95th percentile or significant hyperbilirubinemia) will warrant initiation of phototherapy while those in the Very High Risk Zone (>99th percentile or severe hyperbilirubinemia) will require intensive phototherapy while preparing for exchange transfusion.

## Discussion

This study showed that there was a high correlation between the TsB and TCB levels among Filipino infants. There was a higher correlation between the TsB and the sternal TcB (R^2^ = 0.91) compared with the forehead TcB (R^2^ = 0.88), which could be due to the sternum being covered with clothing, causing less exposure to environmental light. Similarly, a study in Thai infants (26) reflected higher correlation between TsB and sternal TcB compared to forehead TcB. Both studies' results also showed that TcB tends to underestimate TsB values.

There have been concerns about the reliability of TcB among darkly pigmented infants based on how the bilirubinometer uses a light source (xenon) to compute for wavelength differences between skin and subcutaneous tissue (27). However, recent studies have shown TcB readings are not affected by skin color (28). High correlation between TcB and TsB has also been shown among infants of Asian descent such as Japanese (29), Chinese (24), Mongolian (30) and specifically those of Malay descent (darker skin)—Indonesian (31), Myanmar (12) and Thai (26). This study further supports that TcB measurements can reliably estimate total serum bilirubin levels among Filipino infants and as such can be used for bilirubin screening prior to discharge.

The Filipino bilirubin nomogram shows a rapid rise in the bilirubin levels up to 72 HOL and subsequently plateaus thereafter. In this study, there is a two-fold increase from the 3rd to the 6th HOL. There is also an almost two-fold increase from the 12th to the 24th HOL where the steepest rise can be seen in the bilirubin nomogram.

For the 95th percentile TcB values, there is an absolute rise of 8 mg/dL within this 12-hour interval. Subsequently, the rise is 2–3 mg/dl from the 24th to the 48th HOL, and only 1–2 mg/dL from 48th to 72nd HOL and from 72nd to 96th HOL. There is a small (<1 mg/dL) increase from the 96th to 120th HOL. In the Philippines, where majority of infants are discharged at the 24th HOL, bilirubin levels at this time point will expect to increase by 3–5 mg/dL at the 72nd HOL. For those who will be discharged at the 48th HOL, only 1–2mg/dL increase has been noted at the 72nd HOL. These findings will assist in the clinicians' decision making on whether to discharge a jaundiced infant at the 24th HOL.

In this study, the 95th and 99th percentile bilirubin levels at the 72nd HOL are 17 and 19 mg/dL respectively. There is minimal increase (<1 mg/dL) in the bilirubin levels thereafter. Peak bilirubin levels have also been seen around the 72nd HOL in both Caucasians and Asians with higher values found in the latter group (20, 32).

**Figure 5:**
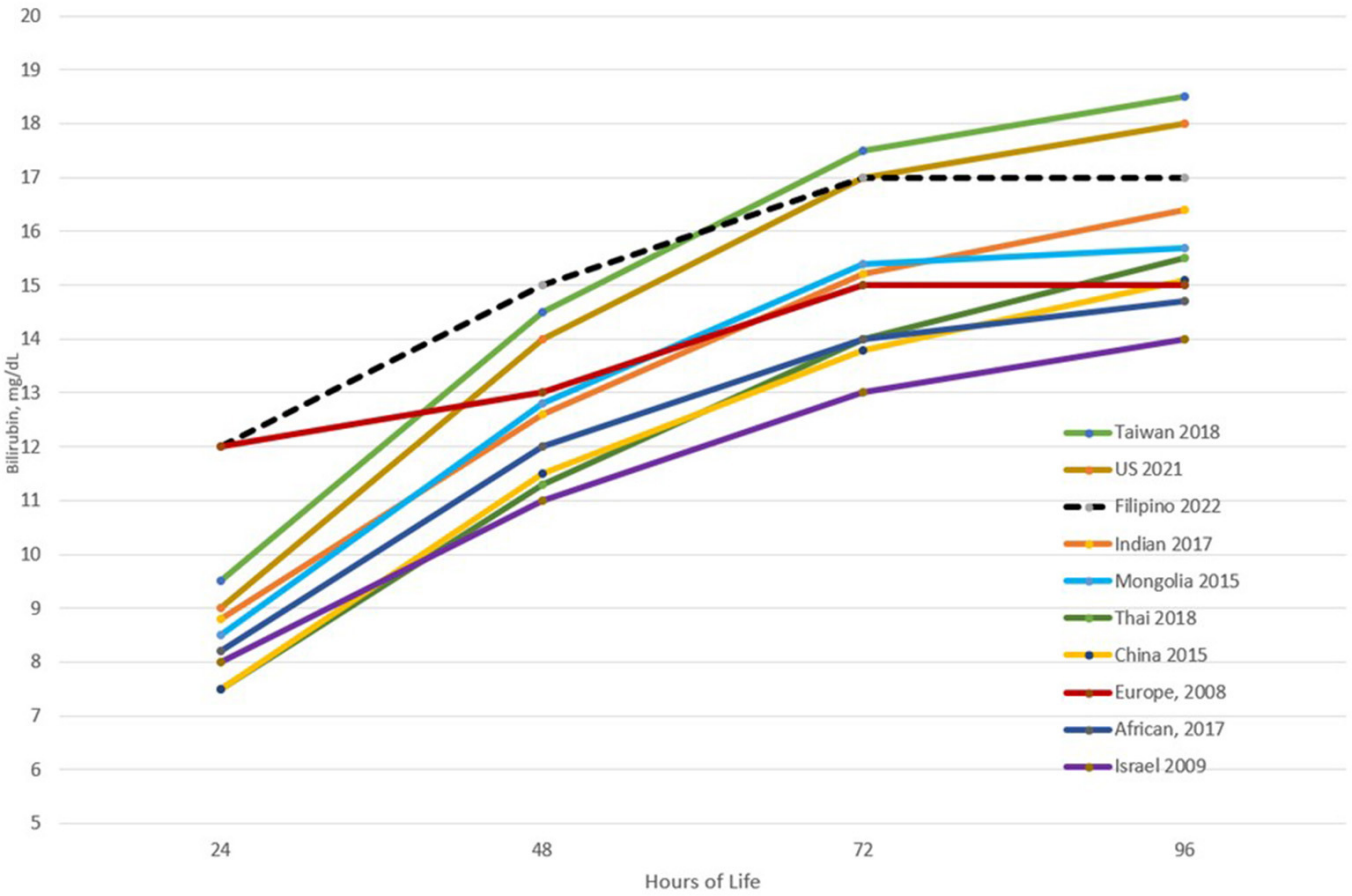
Comparison of the 95th percentile bilirubin values at different time points across different populations.

Upon plotting the 95th percentiles of the bilirubin values from the 24th to the 96th HOL, marked differences from 4 to 5 mg/dL were observed among the different populations/races. Asians were noted to have higher 95th percentile values at all time points. Notably, the new US nomogram also had higher values even if only <2% of the infants were of Asian descent. For the Filipino nomogram, the 95th percentile values were the highest at the 24th to 48th HOL and second highest at the 72nd HOL among the different populations. It plateaus after the 72nd HOL like the Mongolian and European 95th percentile values as opposed to the other populations where the bilirubin values continue to trend upward. This highlighted the importance of developing a nomogram for specific populations due to different bilirubin kinetics. Furthermore, among Filipino infants, close monitoring for the development of significant hyperbilirubinemia should be done in the first 72 hours of life (see Figure 5) (10, 30, 33–39).

The determination of the cut-off for the low-risk zone involved determination of the frequency of developing subsequent SH. In this study, the low-risk zone corresponded to the first quartile where the risk of subsequent development of SH was less than 5%. In the Bhutani bilirubin nomogram, the low-risk zone corresponded to bilirubin values <the 40th percentile which has a <5% risk of developing SH (9, 40). Using the 40th or the 50th percentile as the cut-off for the Filipino nomogram will still have a <5% risk of developing significant hyperbilirubinemia but a 10% risk of reaching bilirubin levels in the HIRZ at the 72nd HOL, where infants with risk factors may be started on phototherapy based on the AAP guidelines (8). Furthermore, TcB values between 25 and 50 percentiles have almost a 20% of shifting to the HIRZ in subsequent time points.

The intermediate risk zone in the Filipino nomogram was the combination of the 2nd and 3rd quartile (25th−75th percentile) since both quartiles correspond to a low risk of developing SH (<5%) but with a higher risk (almost 20% in the first 24th HOL) to shifting to the HIRZ. In the Bhutani chart, there is a designated low intermediate risk zone which is from 41st to 75th percentiles (9). In a review of readmissions due to SH, 28% of the infants who were readmitted were initially in Bhutani's low intermediate risk zone prior to discharge (40). This reiterated that even though bilirubin levels were below the 75th percentiles, close follow up for possible development of significant hyperbilirubinemia should be ensured.

With regards to risk factors for the development of SH at the 72nd HOL, all infants in this study were of East Asian descent (Filipino) which was an established risk factor. Another risk factor such as hemolytic anemia due to ABO/Rh blood incompatibility was not determined since infant blood type was not routinely done in the hospital. Maternal blood type O, *per se*, was not found to be significantly associated with SH in this study.

Filipinos have a high incidence of G6PD deficiency at 1: 50 (21). In a study by Silao et al., 16.7% of Filipinos undergoing phototherapy have G6PD deficiency (21). At the time of this study, G6PD deficiency screening was not yet routinely done in the hospital and thus was not determined. To get more information about G6PD deficiency in the family, mothers were asked if the infant has a sibling with G6PD deficiency, but all mothers were not aware of the condition in the sibling. To determine possible familial reason for hemolysis, report of an elder sibling requiring phototherapy during the neonatal period was not a significant factor for developing SH (*p* = 0.087). Significant risk factors for developing SH at the 72nd HOL were presence of bruising (*p* = 0.001), cephalhematoma (*p* = 0.001), unspecified maternal infection (aside from UTI) (*p* = 0.008) and inadequate prenatal visits (*p* = 0.026).

Inadequate nursing has also been identified as a risk factor for significant hyperbilirubinemia (3). In this study, all the infants were initiated on breastfeeding within 30 mins of life as part of the Essential Intrapartum and Newborn Care protocol. All were exclusively breastfed during the study period. On monitoring of the infants, those who had less frequent feeding, urination, and stooling from the 6th to the 48th HOL had a higher risk of SH. A case control study of infants of Thai descent revealed that early initiation of breastfeeding (1.5 vs. 5.56 h), breastfeeding >8x a day and >10 mins breastfeeding duration were more frequent in the non-jaundice group (41). Lactation counseling starting prenatally and within the first few hours post-delivery focusing on early initiation of breastfeeding, duration, and frequency especially during the first 48 h of life may avert occurrence of SH. Close monitoring of stool and urine frequency, as a surrogate of nursing adequacy, may alert clinicians of any problems with lactation.

In the final Filipino bilirubin nomogram, the AAP risk factors as well as those identified in the study (maternal infection, bruising and cephalhematoma and inadequate nursing) were enumerated as risk factors. Presence of these factors should increase vigilance in monitoring for subsequent development of SH by either delaying discharge for further observation, repeating bilirubin determination after 12–24 h or early clinic follow up 24 h after discharge. Also, presence of the risk factors may lead to earlier initiation of phototherapy at bilirubin values in the high intermediate risk zone (HIRZ) like the AAP recommendations (8).

## Strengths and limitations

This is a relatively large cohort of infants where the bilirubin levels were measured in the first 5 days of life. Feeding patterns in addition to other known risk factors were determined. The limitation of this study is the attrition rate over time due to the early discharge and non-follow up of the infants. Still the total measurements were >1,000 in later time points. Infant blood type and G6PD deficiency determinations were not routinely determined in the hospital. Information from these will better help in the screening and management of the infants.

## Conclusion and recommendation

Transcutaneous bilirubin levels highly correlate with total serum bilirubin levels among Filipino newborn infants. A Filipino nomogram has been developed which showed a rapid rise of bilirubin levels in the first 3 days of life. Bilirubin rise is greatest during the first 24 hours of life, and plateaus after the 72nd hour of life. Presence of bruising and cephalhematoma are important risk factors for the development of SH. Inadequacy of nursing within the first 48 HOL is a modifiable risk factor which can be averted by early lactation counseling and monitoring. The Filipino bilirubin nomogram shows a unique bilirubin kinetics and as such, will better assist the bilirubin screening and subsequent management of Filipino infants.

## Author's note

This work was presented at NIH Anniversary, Bayanihan Hall, Pioneer St. UNILAB, Mandaluyong Metro Manila Philippines in March 2016.

## Data availability statement

The raw data supporting the conclusions of this article will be made available by the authors, without undue reservation.

## Ethics statement

The studies involving human participants were reviewed and approved by NIH Ethical Review Board (Dr. Jacinto V. Mantaring III—Head). Written informed consent to participate in this study was provided by the participants' legal guardian/next of kin.

## Author contributions

MV-U wrote the protocol, supervised the conduct of the study, and also did some of the statistical analysis. HU assisted in the supervision of the conduct of the study and the review of the manuscript. MA computed for the sample size as well as assist in the statistical analysis. All authors contributed to the article and approved the submitted version.

## Funding

This work was supported by NIH-PEER Health Research Fund (Project Code: NIH-2008-018).

## Conflict of interest

The authors declare that the research was conducted in the absence of any commercial or financial relationships that could be construed as a potential conflict of interest.

## Publisher's note

All claims expressed in this article are solely those of the authors and do not necessarily represent those of their affiliated organizations, or those of the publisher, the editors and the reviewers. Any product that may be evaluated in this article, or claim that may be made by its manufacturer, is not guaranteed or endorsed by the publisher.
